# Comparison of GIST and LAMP on the GAW15 simulated data

**DOI:** 10.1186/1753-6561-1-s1-s41

**Published:** 2007-12-18

**Authors:** Xuemei Lou, Silke Schmidt, Elizabeth R Hauser

**Affiliations:** 1 Center for Human Genetics, Duke University Medical Center, 595 South Lasalle Street, Durham, North Carolina 27710, USA; 2 Bioinformatics Research Center, North Carolina State University, Campus Box 7566, Raleigh, North Carolina 27695, USA

## Abstract

After genetic linkage has been identified for a complex disease, the next step is often fine-mapping by association analysis, using single-nucleotide polymorphisms (SNPs) within a linkage region. If a SNP shows evidence of association, it is useful to know whether the linkage result can be explained in part or in full by the candidate SNP. The genotype identity-by-descent sharing test (GIST) and linkage and association modeling in pedigrees (LAMP) are two methods that were specifically proposed to address this question. GIST determines whether there is significant correlation between family-specific weights, defined by the presence of a tentatively associated allele in affected siblings, and family-specific nonparametric linkage scores. LAMP constructs a pedigree likelihood function of the marker data conditional on the trait data, and implements three likelihood ratio tests to characterize the relationship between the candidate SNP and the disease locus. The goal of our study was to compare the two approaches and evaluate their ability to identify disease-associated SNPs in the Genetic Analysis Workshop 15 (GAW15) simulated data. Our results can be summarized as follows: 1) GIST is simple and fast but, as a test of association, did not perform well in the GAW15 data, especially with adjustment for multiple testing; 2) as a test of association, the LAMP-LE test performs best when the linkage evidence is strong, or when there is at least moderate linkage disequilibrium between the candidate SNP and the trait locus. We conclude that LAMP is more flexible and reliable to use in practice.

## Background

The goal of a gene mapping study is to identify genetic variants that predispose to human diseases. For a complex disease, investigators often map the locus of interest first by linkage analysis, which typically results in a large candidate genomic region up to 40 Mb in size. To localize the susceptibility allele more precisely, disease-marker association analyses are performed, using a much denser map of genetic markers within the linkage region. One particular method of association analysis is based on comparing marker allele frequencies between unrelated cases and controls. In this design, only a subset of the samples originally collected for linkage analysis can be used. As an alternative, family-based association methods have been developed. The classic family-based transmission/disequilibrium test was proposed to test for association in the presence of linkage in family trios containing two parents and one affected offspring [1]. This approach has been extended to other family structures [2]. If a single-nucleotide polymorphism (SNP) shows evidence of association, a hypothesis of interest is whether the linkage result can be explained in part or in full by the candidate SNP. The genotype identity-by-descent sharing test (GIST) [3] and linkage and association modeling in pedigrees (LAMP) [4] are two methods that were specifically proposed to address different aspects of this question. The purpose of our study was to evaluate the performance of GIST and LAMP on the simulated Genetic Analysis Workshop 15 (GAW15) data. We used the answers to guide our investigations.

## Methods

### GIST

GIST considers one particular marker allele as tentatively associated with the disease variant, and calculates family-specific weight variables defined by the presence of this allele. The variable has to be defined in such a way that the weight variable and IBD sharing configuration among affected family members at that same locus are uncorrelated if there is no disease-marker association, also called the "unbiased selection scheme". If there is a significant correlation between family-specific weights and family-specific linkage evidence, this suggests that the SNP allele could account in part for the observed linkage signal. GIST calculates three kinds of family-specific weight variables, corresponding to dominant, recessive, or additive inheritance models [3].

Once a weight variable *W *has been defined, the sample correlation coefficient between family weights *W *and family-specific nonparametric linkage (NPL) scores *Z *is computed. Under no disease-marker association, this correlation is expected to be zero and a one-sided test may be performed. A transformation of the correlation coefficient (*X*_*i*_, *i *= *dom*, *rec*, *add*) that is asymptotically standard-normally distributed is used as the test statistic. When we do not know the underlying disease model, an alternative to carrying out all three tests in GIST is to use *X*_*max *_= max(*X*_*dom*_, *X*_*rec*_, *X*_*add*_). The distribution of *X*_*max *_under no disease-marker association is estimated empirically by simulating a large number of ASPs under no linkage for various allele frequencies [3]. The test based on *X*_*max *_should be the most appropriate test because we usually do not know the true genetic model for a complex disease.

### LAMP

LAMP quantifies the degree of linkage disequilibrium (LD) between the candidate SNP and the putative disease locus through joint modeling and estimation of linkage and association parameters. LAMP constructs a likelihood of the marker data conditional on the trait data for a sample of families with disease penetrances and disease-SNP haplotype frequencies as parameters. Model parameters are estimated by maximum likelihood.

Three likelihood ratio tests are proposed to characterize the relationship between the candidate SNP and the disease locus. The first test assesses whether the candidate SNP and the disease locus are linked (LAMP linkage test). The second test is the direct association test, which assesses whether the candidate SNP and the disease locus are in partial LD so that the SNP may account in part for the linkage signal (LAMP-LE test, H_0_: *r*^2 ^= 0). The third test is an indirect association test, which assesses whether there are other variants that can explain the linkage signal (LAMP-LD test, H_0_: *r*^2 ^= 1). If the null hypothesis of complete LD between the SNP and the disease allele is rejected, the SNP does not fully explain the observed linkage signal, and there may be multiple disease variants in this region [4].

### Dataset and analysis

We used all 100 replicates of the simulated GAW 15 family data set to evaluate the power of GIST and LAMP. Each replicate included 1500 nuclear families of size four (two parents and an affected sibling pair (ASP)).

All SNP markers on chromosome 6, 7, 8, 9, 11, 16, and 18 (a total of 3069 SNPs) were analyzed by GIST and LAMP. We also analyzed genotypes at all eight trait loci with LAMP. For GIST, we used the family-specific NPL scores at the location of the maximum multipoint LOD score on each chromosome, using the microsatellite markers [5]. LAMP was run without any flanking markers. To compare the LAMP results to standard methods for linkage and association analysis, we also calculated the average multipoint LOD score at the SNP closest to the trait locus, using the SNP markers, and performed family-based association analysis of each SNP with the pedigree disequilibrium test (PDT) [2]. LD analysis for 50 SNP markers surrounding each trait locus was performed with GOLD, using the Replicate 1 data only [6].

### Power calculation

The power to reject the null hypothesis of GIST and LAMP-LE was defined in two different ways: if we considered all SNP markers in the region defined by the true trait locus ±5 cM as the correct candidates, the power was estimated by the proportion of replicates with at least one *p*-value above a threshold value. If we only considered the SNP closest to the trait locus as the candidate SNP, the power was estimated by the proportion of replicates in which the *p*-value at this SNP was above a threshold value.

The threshold value was set differently for each test. Because chromosome 7 does not contain any trait loci, the analysis results (*p*-values) obtained for this chromosome across the 100 replicates define a null distribution for the hypothesis of "no linkage and no association". Because it was previously shown that linkage and association test statistics are independent under this null hypothesis [7], this hypothesis implies the null hypothesis of GIST (no correlation between family-specific NPL-scores and weights derived from "non-associated" genotypes). Therefore, this is the relevant null distribution for the GIST combined test and LAMP-LE test. The fifth percentile value of the chromosome 7 *p*-value distribution for each test was set as the threshold value (false positive rate α = 0.05).

To derive a null distribution for the LAMP-LD test (*r*^2 ^= 1), we analyzed genotypes at the actual trait loci. Due to the complexity of the GAW15 simulation model, we do not have the perfect null situation. Because a basic assumption of LAMP is the presence of only one disease variant in the region, we decided not to use the trait loci on chromosome 6 and 9. Despite the fact that none of loci A, B, E, and F are "pure" disease susceptibility loci, we used these loci as the best guess approximation for the null distribution for the LAMP-LD test.

## Results

### Characteristics of trait loci and surrounding region

To evaluate different methods, it is useful to gain a better understanding of the simulated data first. Table 1 lists the risk allele frequency at each trait locus, pairwise *r*^2 ^values between the closest SNP marker and each trait locus, the number of markers in the ±5 cM region, the maximum *r*^2 ^between SNP and the trait locus in the ±5 cM region, and the average maximum NPL-based LOD score for each chromosome. Pairwise *r*^2 ^values among the 50 SNP markers and the trait locus are not shown, but most of them were less than 0.05. In other words, there is not much LD among the markers in the ±5 cM region.

**Table 1:** Characteristics of trait loci and surrounding region

	Trait loci			Closest SNP			±5 cM region		Average max NPL (SD) on chromosome
				
Chr	Locus	cM	Risk allele frequency	Marker	cM	*r*^2^	Number of markers	Maximum *r*^2^	
A	16	26.29	0.3658	SNP16_31	26.31	0.008	15	0.016	0.60(0.60)
B	8	170.9	0.3992	SNP8_442	167.6	0.002	10	0.006	0.53(0.47)
C	6	49.46	0.8688	SNP6_153	49.46	0.551	48	0.953	62.99(7.76)
D	6	54.57	0.0418	SNP6_162	54.62	0.958	22	0.958	62.99(7.76)
E	18	94.27	0.3797	SNP18_269	94.22	0.145	26	0.145	1.21(0.91)
F	11	115.29	0.6123	SNP11_389	115.28	0.94	41	0.94	0.84(0.63)
G	9	49.40	0.0938	SNP9_185	49.32	0.007	48	0.014	0.56(0.48)
H	9	51.41	0.1663	SNP9_192	51.40	0	45	0.011	0.56(0.48)
Null	7								0.56(0.48)

From Table 1, we can see that locus C and F have a major risk allele. Locus D has a rare minor risk allele with frequency less than 0.05. *r*^2 ^values between locus C, D, E, and F and the closest SNP are over 0.1, while *r*^2 ^values between locus A, B, G, and H and the respective closest SNP are practically 0. The maximum *r*^2 ^values in the ±5 cM region around locus A, B, G, and H are all less than 0.02. The linkage evidence for chromosome 6 is overwhelmingly high, but only modest NPL-based LOD scores were obtained for chromosomes 11 and 18. For chromosomes 8, 9, and 16, the NPL-based LOD scores are close to those for the "null" chromosome 7.

### Power comparison between GIST and LAMP tests

The empirical 5% threshold value obtained from the chromosome 7 analysis was 0.0515 for the GIST combined test, and 0.04 for LAMP-LE. The empirical 5% threshold values for LAMP-LD from locus A, B, E, and F were 0.08, 0.04, 0.02, and 0.14. Because only 100 replicates were available, these values were consistent, with a 0.05 nominal *p*-value. Using these empirical thresholds, we calculated the power to reject the null hypotheses of GIST and LAMP-LE at the closest SNP (Table 2). The power of the GIST combined test is low for each trait locus, while LAMP-LE has 100% power to reject the null hypothesis for the closest SNP when there is at least moderate LD between the candidate SNP and the trait locus, such as locus E. Like GIST, the LAMP-LE test has similarly low power for locus A, B, G and H when the *r*^2 ^values are less than 0.01.

**Table 2:** Power comparison of GIST and LAMP at the SNP closest to trait locus

Locus	Chr	GIST Combined Test (%)	LAMP-LE Test (%)	LAMP-LD Test (%)
A	16	4	4	5
B	8	10	8	2
C	6	4	100	74
D	6	2	100	68
E	18	7	100	6
F	11	4	100	3
G	9	4	2	2
H	9	10	6	0

The power comparison of GIST and LAMP-LE across a ±5 cM region is shown in Table 3. Using the same threshold values as for Table 2, we calculated the power with and without adjustment for multiple testing. The Bonferroni correction for multiple testing corresponds to dividing the respective thresholds by the number of markers in the 10-cM region surrounding each locus. In Table 3, the unadjusted power of the GIST combined test is reasonably high for all trait loci, but after adjusting for multiple testing, it is similarly low as in Table 2. The LAMP-LE test still has almost 100% power to reject linkage equilibrium when there is at least moderate LD (i.e., *r*^2 ^≥ 0.145), even with adjustment for multiple testing, but, as expected, has low power when there is no LD, e.g., locus A, B, G, and H, as in Table 2. The power of the LAMP-LE test increases when *r*^2 ^increases.

**Table 3:** Power comparison of GIST and LAMP within ±5 cM of trait locus

		GIST combined test (%)		LAMP-LE test (%)		LAMP-LD test (%)	
				
Locus	Chr	Unadjusted	Adjusted	Unadjusted	Adjusted	Unadjusted	Adjusted
A	16	48	3	59	7	29	0
B	8	46	6	44	7	14	1
C	6	82	3	100	100	100	100
D	6	60	4	100	100	100	100
E	18	75	3	100	99	60	5
F	11	93	6	100	100	50	4
G	9	85	7	80	1	34	0
H	9	87	5	87	3	35	0

The pattern of the power estimates for the LAMP-LD test is similar in Tables 2 and 3, and consistent with expectations. The power decreases when *r*^2 ^increases, and also depends on the magnitude of the linkage evidence. For locus C and D, both located on chromosome 6 and showing very strong evidence of linkage, the power to reject the the null hypothesis of complete LD with a single susceptibility variant is high, even though there is almost complete LD with the closest SNP (*r*^2 ^= 0.94). Locus G and H are also located on the same chromosome, but they show little evidence of linkage and hence the power to reject complete LD is low. For locus A and B, LAMP-LD has low power to reject complete LD, presumably also because of little linkage evidence. Because locus E has a lower *r*^2 ^value with the closest SNP than locus F, and they both have moderate linkage evidence, it makes sense that the power to reject complete LD is higher for locus E than locus F.

### Comparison of MERLIN and LAMP linkage test, PDT and LAMP-LE test

We compared the results from the LAMP linkage test with a standard linkage analysis using MERLIN, and also compared results from the LAMP-LE association test with PDT (data not shown). These comparisons suggest that the linkage and association tests from LAMP for these family structures are very similar to the linkage test implemented in MERLIN and the family-based association test implemented in PDT, respectively.

## Discussion

We have completed GIST and LAMP analysis on eight totally different trait loci for rheumatoid arthritis simulated in GAW15 using SNPs that mimic a 10 K SNP chip set and microsatellite markers. Our results from applying linkage and association tests to the data are consistent with the genetic effects and levels of LD for each chromosome.

GIST can only be applied to affected sibship data and considers only genotypes at the tentatively associated SNP, without incorporating flanking markers. In theory, GIST can detect association in regions with little overall evidence for linkage. Our results show that the GIST combined test performs poorly even in cases which would seem to be favorable for detecting an associated SNP (both linkage and LD). A possible reason for the failure of GIST to detect associated alleles is the multiple loci interacting to affect the RA hazard, and the overwhelming genetic heterogeneity, perhaps even within-family heterogeneity. Because the linkage evidence is very modest on chromosomes 11, 16, and 18 (average LOD scores < 1), there is also little power to detect association to the disease alleles.

LAMP can be applied to general pedigree data, including affected and unaffected individuals, and can incorporate flanking markers. Our study shows that LAMP-LE works well when there is at least moderate LD between marker and disease variant, even when *r*^2 ^values are as low as 0.145. The major disadvantage of LAMP is the speed. For large and sparsely genotyped pedigrees, LAMP can be painfully slow and such pedigrees must be trimmed or discarded in practice.

## Conclusion

GIST is simple and fast once family-specific NPL scores and weight variables have been computed, but it did not perform well in the GAW15 simulated data. LAMP is more flexible and more powerful, but much slower. LAMP seems to work particularly well when there is at least moderate LD between the candidate SNP marker and the trait locus.

## Competing interests

The author(s) declare that they have no competing interests.
